# Whole Genome Expression Profiling and Signal Pathway Screening of MSCs in Ankylosing Spondylitis

**DOI:** 10.1155/2014/913050

**Published:** 2014-12-03

**Authors:** Yuxi Li, Peng Wang, Zhongyu Xie, Lin Huang, Rui Yang, Liangbin Gao, Yong Tang, Xin Zhang, Jichao Ye, Keng Chen, Zhaopeng Cai, Yanfeng Wu, Huiyong Shen

**Affiliations:** ^1^Department of Orthopedics, Sun Yat-sen Memorial Hospital, Sun Yat-sen University, No. 107 Yanjiang Road West, Guangzhou 510120, China; ^2^Center for Biotherapy, Sun Yat-sen Memorial Hospital, Sun Yat-sen University, No. 107 Yanjiang Road West, Guangzhou 510120, China

## Abstract

The pathogenesis of dysfunctional immunoregulation of mesenchymal stem cells (MSCs) in ankylosing spondylitis (AS) is thought to be a complex process that involves multiple genetic alterations. In this study, MSCs derived from both healthy donors and AS patients were cultured in normal media or media mimicking an inflammatory environment. Whole genome expression profiling analysis of 33,351 genes was performed and differentially expressed genes related to AS were analyzed by GO term analysis and KEGG pathway analysis. Our results showed that in normal media 676 genes were differentially expressed in AS, 354 upregulated and 322 downregulated, while in an inflammatory environment 1767 genes were differentially expressed in AS, 1230 upregulated and 537 downregulated. GO analysis showed that these genes were mainly related to cellular processes, physiological processes, biological regulation, regulation of biological processes, and binding. In addition, by KEGG pathway analysis, 14 key genes from the MAPK signaling and 8 key genes from the TLR signaling pathway were identified as differentially regulated. The results of qRT-PCR verified the expression variation of the 9 genes mentioned above. Our study found that in an inflammatory environment ankylosing spondylitis pathogenesis may be related to activation of the MAPK and TLR signaling pathways.

## 1. Introduction

Ankylosing spondylitis (AS) is a chronic, immune-mediated inflammatory disease characterized by inflammatory back pain and enthesis [1]. To date, several hypotheses have been proposed to describe the pathogenic mechanism behind this disease, including genetic susceptibility of HLA-B27 [2] and ERAP1 [3], infection [4], and environmental factors [5]. However, none of these hypotheses can fully account for the pathogenesis of ankylosing spondylitis. Recent evidence has increasingly suggested that autoimmune disorders may be involved in the onset and development of this disease [6].

It has been recently demonstrated that mesenchymal stem cells (MSCs) possess an immunosuppressive capability to inhibit Th17 cells and induce Treg subpopulations of CD4+ T cells [7, 8]. In 2011, our research showed that compared to healthy donors, the immunomodulatory ability of MSCs was reduced in AS patients. This was manifested by an increase in Th17 cells and a reduction in Treg cells in CD4+ T cell subgroups after a mixed lymphocyte reaction (MLR) [9]. Furthermore, our clinical trial has also suggested that infusion of MSCs is a feasible, safe, and promising treatment for AS patients [10]. Therefore, we believe that the modulatory function of MSCs can play a significant role in improving disease condition or clinical symptoms in AS patients and that immunoregulatory dysfunction of MSCs may play a critical role in the pathogenesis of AS.

Recently, several researchers carried out whole genome expression profiling analyses comparing AS patients to healthy donors [11–13]. These studies found that AS has a strong association with HLA-B27, and other non-HLA susceptibility genes, such as IL23R and ERAP1 [14, 15]. However, these genetic variations could not fully account for the pathogenesis of ankylosing spondylitis. For example, HLA-B27 accounts for only ~45% of the genetic risk for AS. Therefore, in this study we focused mainly on the significant role of MSCs in AS pathogenesis in order to provide a new viewpoint on this inflammatory disease. Our study investigated whether MSCs grown in vitro from AS patients exhibit gene expression differences in normal culture media or in an inflammatory environment, as compared to healthy controls. We found that mimicking an inflammatory environment can activate the MAPK and TLR signaling pathways in MSCs derived from AS patients, thereby upregulating inflammatory gene expression. This data provides suggestive clues in the exploration of the pathogenic mechanism behind ankylosing spondylitis.

## 2. Materials and Methods

### 2.1. Patients and Controls

The present study was approved by the Ethics Committee of the Sun Yat-sen Memorial Hospital of Sun Yat-sen University, Guangzhou, China. From June 2012 to December 2012, twelve healthy donors (HD, 9 men and 3 women) with an average age of 22.1 years and twelve AS patients (10 men and 2 women) with an average age of 21.9 years were enrolled in this study (Table 1). The diagnoses of AS patients were all performed according to the ASAS classification criteria [16]. In addition, all patients involved were diagnosed for the first time and remained in active stage (all Bath Ankylosing Spondylitis Disease Activity Index ≥ 4).

### 2.2. Isolation and Preparation of MSCs

After being informed about scientific significance, possible risks and complications, and the treatment measures for bone marrow aspirations, all donors and patients signed the informed consent and were aspirated by our skilled allied health professionals in strict accordance with the international standardized procedure. Bone marrow MSCs were isolated from the bone marrow aspirates by density gradient centrifugation as previously described by our work and that of others [9, 17]. After identifying MSC immunophenotype markers by flow cytometry, passages three to five were used for the experiments described below. Both HD and AS groups were separated into two subgroups: MSCs cultured in normal media (Norm) and MSCs cultured in an inflammatory environment (Infla) which was created by adding TNF-*α* (10 ng/mL) and IFN-*γ* (10 ng/mL) into normal culture media for four hours. Five representatives of cultured MSCs from HD and AS patients were randomly chosen, cultured in both types of media, and further analyzed. Twenty samples were chosen to complete whole genome expression profiles: 5 HD-Norm, 5 HD-Infla, 5 AS-Norm, and 5 AS-Infla samples.

### 2.3. Total RNA Extraction and Quality Control

1 mL Trizol (Invitrogen, Carlsbad, USA) was added to lyse the collected cells. Total RNA was extracted using the phenol/chloroform method and salt was washed away with 70% alcohol. After air-drying at 15~30°C, the appropriate amount of RNase-free water was added to dissolve RNA. The integrity and concentration of RNA were assessed to ensure accuracy and reliability. Total RNA from each sample was quantified using a NanoDrop 1000, and RNA integrity was assessed by standard denaturing agarose gel electrophoresis. About 5 *μ*g total RNA from each sample was used for labeling and array hybridization following these steps: (1) Reverse transcription by Invitrogen Superscript ds-cDNA synthesis kit; (2) ds-cDNA labeling with NimbleGen one-color DNA labeling kit; (3) array hybridization using the NimbleGen Hybridization System, followed by washing with the NimbleGen wash buffer kit; (4) array scanning using the Axon GenePix 4000B microarray scanner (Molecular Devices Corporation).

### 2.4. Microarray Scanning and Data Analysis

Scanned images were then imported into NimbleScan software (version 2.5) for grid alignment and expression data analysis. Data were normalized by quantile normalization and the robust multichip average (RMA) algorithm included in the NimbleScan software. Probe level files and gene level files were generated after normalization. The twenty gene level files were imported into Agilent GeneSpring GX software (version 11.5) for further analysis. After GeneSpring quantile normalization, differentially expressed genes were identified using Volcano Plot filtering. KEGG pathway analysis and GO analysis were applied to determine the roles of these differentially expressed genes in biological pathways or GO terms. Finally, the post hoc Bonferroni test was used to correct for multiplicity since a large number of false positive *P* values were observed. *P* values <0.01 were considered significant.

### 2.5. Quantitative Real-Time Polymerase Chain Reaction (qRT-PCR)

After MSCs were cultured in inflammatory environment mentioned above, total RNA was extracted using Trizol reagent (Life Technologies Inc., USA). Synthesis of cDNA (RT-PCR) was carried out using the SuperScriptH III First-Strand Synthesis System (Invitrogen) according to the manufacturer's instructions. The quantitative real-time PCR (qRT-PCR) analyses were performed in a Light Cycler 480 Real-Time PCR System (Roche, Basel) using a SYBR Premix Ex Taq II kit (Takara, Otsu). The program for quantitative-PCR assay was 95°C for 30 s, followed by 40 cycles of 95°C for 5 s and 60°C for 20 s. The mean mRNA levels were calculated from triplicate analyses of each sample and melting curve analysis was performed to confirm the specific amplification of the targets. The relative amount or fold change of the target genes was normalized according to the expression of the housekeeping gene*β*-actin. Parts of the primer sequences used in the real-time PCR assay are as follows:

 
*β*-actin: 5′-CATGTACGTTGCTATCCAGGC-3′ (Forward) and 5′-CTCCTTAATGTCACGCACGAT-3′ (Reverse);
 
*GNA12*: 5′-CCGCGAGTTCGACCAGAAG-3′ (Forward) and 5′-TGATGCCAGAATCCCTCCAGA-3′ (Reverse);
 
*DUSP1*: 5′-AGTACCCCACTCTACGATCAGG-3′ (Forward) and 5′-GAAGCGTGATACGCACTGC-3′ (Reverse);
 
*MAPK11*: 5′-CTGAACAACATCGTCAAGTGCC-3′ (Forward) and 5′-CATAGCCGGTCATCTCCTCG-3′ (Reverse);
 
*RAC1*: 5′-ATGTCCGTGCAAAGTGGTATC-3′ (Forward) and 5′-CTCGGATCGCTTCGTCAAACA-3′ (Reverse);
  
*MAPK3*: 5′-CTACACGCAGTTGCAGTACAT-3′ (Forward) and 5′-CAGCAGGATCTGGATCTCCC-3′ (Reverse);
 
*TRAF3*: 5′-CAGACTAACCCGCCGCTAAAG-3′ (Forward) and 5′-GATGCTCTCTTGACACGCTGT-3′ (Reverse);
 
*IFNAR1*: 5′-AACAGGAGCGATGAGTCTGTC-3′ (Forward) and 5′-TGCGAAATGGTGTAAATGAGTCA-3′ (Reverse);
 
*IL6*: 5′-ACTCACCTCTTCAGAACGAATTG-3′ (Forward) and 5′-CCATCTTTGGAAGGTTCAGGTTG-3′ (Reverse);
 
*IFNA1*: 5′-GCCTCGCCCTTTGCTTTACT-3′ (Forward) and 5′-CTGTGGGTCTCAGGGAGATCA-3′ (Reverse).

## 3. Results

### 3.1. Identification of MSC Immunophenotype

**Figure 1:**
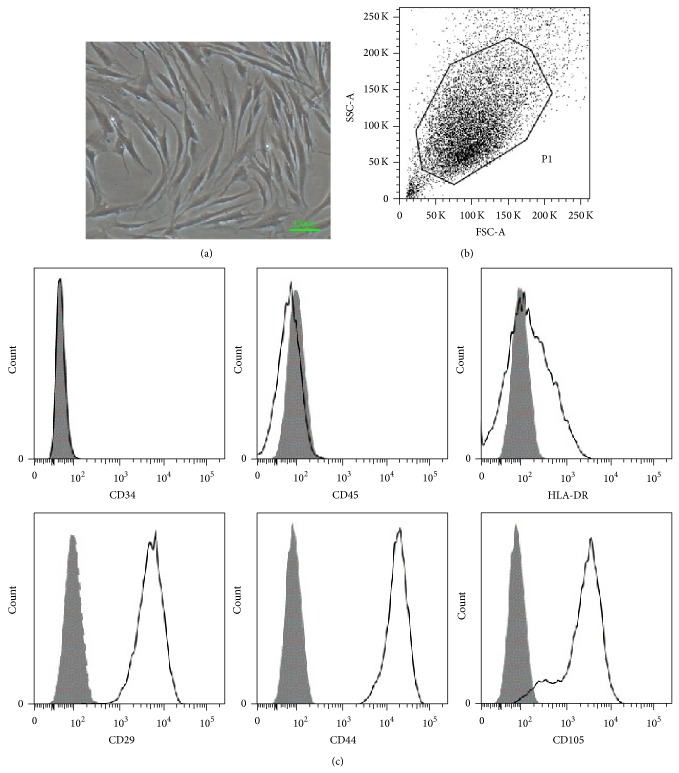
Immunophenotype and differentiation of MSCs. (a) Under the 40x microscope, spindle-shaped MSCs grow like intersecting bundles. (b) Dot plot data showing all (nongated) cells as well as gated hMSC (P1). (c) MSCs were harvested, labeled with antibodies against immunophenotype, and analyzed by flow cytometry. MSCs expressed CD29, CD44, and CD105 but not CD34, CD45, and HLA-DR.

MSCs were obtained from bone marrow aspirates of both healthy volunteers and AS patients. MSCs were cultured and purified to homogeneity according to the culture method described in our previous work [9].  Figure 1(a) shows adherent MSCs growing like intersecting spindle bundles. In addition, immunophenotyping was performed by flow cytometry. Dot plots showing all (ungated) cells as well as gated MSCs are displayed in Figure 1(b). MSCs constitutively expressed CD29, CD44, and CD105 but not CD34, CD45, or HLA-DR (Figure 1(c)).

### 3.2. Microarray Hybridization and Data Analysis

**Figure 2:**
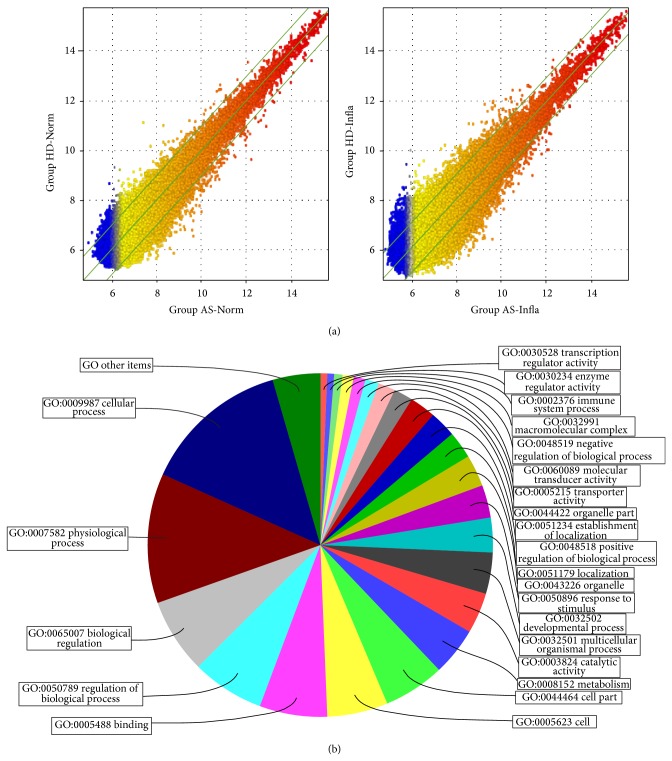
Whole genomic expression analysis of MSCs in AS patients. (a) shows the scatter plot of differentially expressed genes of MSCs from both healthy donors and AS patients, respectively, in both normal and inflammatory culture environment. The values of *x* and *y* axes in the scatter plot are the normalized signal values of each sample (log2 scaled). The green lines are fold change lines (the default fold change value given is 2.0). Genes above the top green line and below the bottom green line indicated more than 2.0-fold change of genes between two compared arrays. (b) shows the pie chart of gene ontology (GO) analysis of results of the differentially expressed genes of MSCs between group AS-Infla and group HD-Infla. Among the 1590 genes, 892 (56.10%) participated in biological process, 461 (28.99%) played a role in molecular function, and 237 (14.91%) associated with cellular component.

The experimental system was stable and fluorescent signal intensity was strong and homogenous. When cultured under inflammatory conditions, the number of differentially expressed genes in MSCs from AS patients was increased (Figure 2(a)). There were 676 differentially expressed genes in the AS-Norm group, of which 354 were upregulated and 322 were downregulated as compared to the HD-Norm group. 1767 genes were differentially expressed in the AS-Infla group, 1230 upregulated and 537 downregulated, as compared to the HD-Infla group. We used GO (gene ontology) to analyze these differentially expressed genes within three domains (Figure 2(b)).

In the biological processes domain, we found 79 significant functional description nodes for group AS-Norm, which were mainly related to intracellular signaling cascades, positive regulation of cellular metabolic processes, regulation of molecular function, positive regulation of macromolecule metabolic processes, positive regulation of cellular biosynthetic processes, and positive regulation of metabolic processes. There were 134 functional description nodes (*P* < 0.01) in group AS-Infla versus group BD-Infla, which were mainly related to mRNA metabolic processes, nucleobase, nucleoside, nucleotide and nucleic acid metabolic processes, cell cycle, mitotic cell cycle, gene expression, and nitrogen compound metabolic processes (Table 2).

In the cellular component domain, 8 significant functional description nodes were found for the AS-Norm group, compared to the BD-Norm group, which were mainly located in the extracellular region, cyclin-dependent protein kinase holoenzyme complex, extracellular region part, keratin filament, neuron projection, cytoplasmic microtubule, extracellular space, and apical plasma membrane. In contrast, compared to group BD-Infla, there were 44 significant functional description nodes for the AS-Infla group, which were mainly located in the intracellular part, intracellular membrane-bounded organelle, membrane-bounded organelle, intracellular organelle lumen, and organelle lumen (Table 3).

In the molecular function domain, when compared to group BD-Norm there were 12 significant functional description nodes for the AS-Norm group, such as protein tyrosine/serine/threonine phosphatase activity, growth factor activity, fibroblast growth factor receptor binding, and receptor signaling protein activity. Simultaneously, 20 significant functional description nodes (*P* < 0.01) were found for group AS-Infla versus group BD-Infla, which included protein binding, nucleic acid binding, structure-specific DNA binding, RNA binding, and hexosaminidase activity (Table 3).

### 3.3. Screening of Signaling Pathways Related to AS

**Figure 3:**
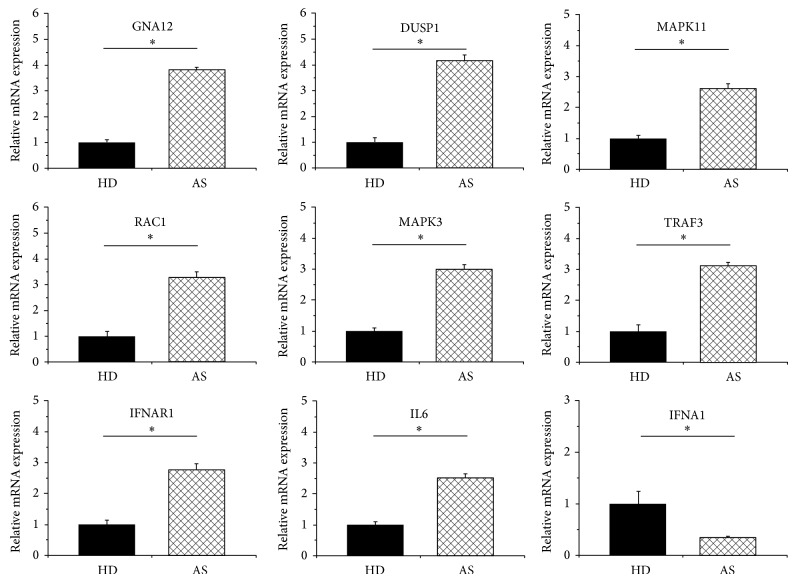
Verification of differentially expressed genes of MSCs in AS patients cultured in inflammatory environment. RNA was isolated from MSCs of HD (*n* = 5) and AS patients (*n* = 5) after MSCs were cultured in inflammatory environment mimicked by TNF-*α* (10 ng/mL) and IFN-*γ* (10 ng/mL). Comparison of the levels of differentially expressed genes in MSCs from both groups was based on quantitative real-time polymerase chain reaction (qRT-PCR). Beta-actin was used as an internal control. The results showed that when grown in inflammatory culture medium, 9 of 22 differentially expressed genes from whole genomic expression analysis in AS patients were truly differentially expressed. These genes include GNA12, DUSP1, MAPK11, RAC1, MAPK3, TRAF3, IFNAR1, IL6, and IFNA1. The symbol “∗” represents *P* < 0.05.

KEGG pathway analysis was used to discover key signaling pathways and relationships between differentially expressed genes. In the inflammatory environment, the two highest scores of indicators in the AS group were hsa04010: mitogen-activated protein kinase (MAPK) signaling pathway, and hsa04620: toll-like receptor (TLR) signaling pathway (Table 4). In the MAPK pathway, there were 14 differentially expressed genes (*P* < 0.05): DUSP1, GNA12, RAC1, MRAS, ELK1, ATF4, MAPK3, CDC25B, STMN1, MAX, TGFBR2, MAPK11, CACNB3, FGF8, and RASA2 (Table 5). In the TLR pathway, there were 8 key genes with a significant difference in expression (*P* < 0.05): RAC1, TRAF3, MAPK3, IFNAR1, TICAM1, IL6, MAPK11, and IFNA1 (Table 5). However, there were only two differentially expressed genes (CACNA2D3 and PPM1B) in the MAPK pathway and one key gene (TLR6) in the TLR pathway in the AS group cultured in normal media, both with an insignificant KEGG analysis score (*P* > 0.05). The results of qRT-PCR verified the expression variation of such genes mentioned above, including GNA12, DUSP1, MAPK11, RAC1, MAPK3, TRAF3, IFNAR1, IL6, and IFNA1 (Figure 3).

## 4. Discussion

Ankylosing spondylitis is a type of chronic inflammatory arthritis. Our previous work demonstrated its pathogenesis may be associated with immunomodulatory dysfunction of MSCs in AS patients [9]. The pathogenesis of dysfunctional immunoregulation of MSCs in AS is thought to be a complex process involving multiple genetic alterations. In this study we focused our interest on the difference in MSCs between AS patients and healthy donors and carried out whole genome expression profiling. Our study showed that there were 676 genes differentially expressed between the AS-Norm group and HD-Norm group, 354 upregulated and 322 downregulated. 1767 genes were differentially expressed between the AS-Infla group and HD-Infla group, 1230 upregulated and 537 downregulated. Furthermore, we found that when cultured in an inflammatory environment, MSCs from AS patients suggested an activation of the MAPK and TLR signaling pathways.

By GO analysis, differentially expressed genes involved in biological processes were mainly related to mRNA metabolic processes, nucleobase, nucleoside, nucleotide and nucleic acid metabolic processes, cell cycle, mitotic cell cycle, gene expression, and nitrogen compound metabolic processes. In addition, differentially expressed genes involved mainly in molecular functions were mainly related to protein binding, nucleic acid binding, structure-specific DNA binding, RNA binding, and hexosaminidase activity. Furthermore, KEGG pathway analysis showed that MAPK and TLR signaling pathways were significantly associated with MSCs from AS patients in an inflammatory environment.

Some early research has confirmed that TNF induces a response in MSCs by activating MAPK-dependent mechanisms [18]. In addition, whole blood transcriptional profiling of AS patients has also revealed inflammatory candidate genes including MAPK [19]. We have to consider that, under inflammatory conditions, the MAPK signaling pathway may play a key role in MSCs of AS patients. In this study 14 MAPK signaling genes were differentially expressed, including GNA12, DUSP1, MAPK11, RAC1, MRAS, ELK1, ATF4, MAPK3, CDC25B, STMN1, MAX, TGFBR2, CACNB3, FGF8, and RASA2 (*P* < 0.01). Some of these genes have been reported to have a relationship with the immune system or even with inflammatory autoimmune diseases. For example, GNA12 has been demonstrated to play a key role in barrier function in the development of ulcerative colitis [20]. DUSP1 (also referred to as MKP1) has been shown to be a critical negative regulator of inflammatory cytokines [21]. Increased expression of DUSP1 in group AS-Infla may be the result of systemic inflammation in AS patients. Moreover, RAC1 activation can be directly induced by IL17A [22] and RAC1 inhibitory peptide has been found to suppress T cell activation and autoantibody production in autoimmune disease [23]. MAPK11 (also referred to as p38BETA) is one of the four members of the p38MAPK family (p38alpha, p38beta, p38gamma, and p38delta). A large body of evidence now indicates that p38MAPK activity is critical for normal immune and inflammatory responses. The p38MAPK pathway is a key regulator of proinflammatory cytokine biosynthesis at both the transcriptional and translational levels, making components of this pathway attractive potential targets for the treatment of autoimmune and inflammatory diseases [24]. Furthermore, p38MAPK inhibitors have been verified to be efficacious in animal models of rheumatoid arthritis, such as collagen-induced arthritis in the mouse and adjuvant-induced arthritis in the rat [25, 26]. Our study has reported that the above-mentioned genes were significantly upregulated in MSCs from AS patients, suggesting a role in the pathogenesis of ankylosing spondylitis. However, the relationship between these differentially expressed genes and ankylosing spondylitis has not been previously reported.

It is well known that upregulation of TLR depends on MAPK signaling [27]. Our present study found that eight key genes from the TLR signaling pathway (including RAC1, TRAF3, MAPK3, IFNAR1, TICAM1, IL6, MAPK11, and IFNA1) were significantly differentially expressed (*P* < 0.01). Some previous studies have reported that IL6 may participate in the pathogenesis of rheumatoid arthritis (RA), and several clinical trials have been carried out evaluating safety and efficacy of Tocilizumab—an anti-IL6 antibody—in RA patients [28]. Increased IL6 expression in AS patients observed in our study may provide a helpful clue in illustrating its pathogenesis. In addition, tumor necrosis factor receptor-associated factor 3 (TRAF3) is a negative regulator of IL17 receptor (IL17R) signaling. TRAF3 suppresses IL17-induced NF-*κ*B and mitogen-activated protein kinase activation and the subsequent production of inflammatory cytokines and chemokines [29]. Increased expression of TRAF3 observed in group AS-Infla may be due to persistent inflammation in AS patients. Moreover, Kole et al. demonstrated that IFN-1 plays an essential role in producing anti-inflammatory cytokines and maintaining intestinal T cell homeostasis by both limiting effector T cell expansion and promoting Treg stability [30]. Using a knockout mouse model, De Weerd et al. found that lipopolysaccharide-induced sepsis was ameliorated in Ifnar1^−/−^ mice but not Ifnar2^−/−^ mice, which suggests IFNAR1-IFN-*β* signaling plays a pathological role in inflammatory disease [31]. This study found that IFNA1 was significantly reduced while IFNAR1 was enhanced in MSCs derived from AS patients cultured in an inflammatory environment, which accounts for the increased proinflammatory ability and reduced anti-inflammatory status of AS patients.

## 5. Conclusions

Our present study confirmed that the pathogenesis of AS stems from multiple gene interactions and key signaling pathway activation. Our study found that an inflammatory environment may activate the MAPK and TLR signaling pathways in MSCs derived from AS patients and causes upregulation of inflammatory gene expression. This data provides suggestive clues in exploring the pathogenesis of ankylosing spondylitis. Finally, further verification and validation of these differentially expressed genes in the pathogenesis of AS will be needed in future research.

## Supplementary Material

Supplementary Table 1: GO analysis of ankylosing spondylitis with different biological processes related to gene.

## Figures and Tables

**Table 1:** Demographic data and disease characteristics of enrolled patients and healthy donors.

Items	AS	HD
Numbers	12	12
Sex		
Men	10	9
Women	2	3
Age (years)	22.9 (20–30)	23.1 (21–31)
Body weight (Kg)	63.3 (58–65)	63.5 (60–66)
Disease duration from diagnosis (months)	33.9 (28–39.6)	0
HLA-B27	11 (91.7%)	0
History of extra-axial involvement (number of patients)		
Uveitis	0	0
Psoriasis	0	0
Dactylitis	2 (16.7%)	0
Inflammatory bowel disease	1 (8.3%)	0
Enthesitis	5 (41.7%)	0

**Table 2:** GO analysis of ankylosing spondylitis with different biological processes related to gene.

Category	GO ID	Term	Count of genes	Percent of count of genes	Fold enrichment	*P* value
GOTERM_BP	GO:0002376	Immune system process	50	12.69	1.7	0.00016
GOTERM_BP	GO:0002520	Immune system development	19	4.82	2.03	0.00285
GOTERM_BP	GO:0003006	Reproductive developmental process	14	3.55	2.05	0.00898
GOTERM_BP	GO:0006325	Chromatin organization	21	5.33	1.95	0.00276
GOTERM_BP	GO:0006816	Calcium ion transport	13	3.3	2.71	0.00107
GOTERM_BP	GO:0006950	Response to stress	64	16.24	1.35	0.00765
GOTERM_BP	GO:0006996	Organelle organization	54	13.71	1.4	0.00669
GOTERM_BP	GO:0007154	Cell communication	71	18.02	1.59	0.00005
GOTERM_BP	GO:0007165	Signal transduction	108	27.41	1.24	0.00756
GOTERM_BP	GO:0007242	Intracellular signaling cascade	73	18.53	1.7	0
GOTERM_BP	GO:0007243	Protein kinase cascade	25	6.35	1.71	0.00663
GOTERM_BP	GO:0007275	Multicellular organismal development	100	25.38	1.28	0.00422
GOTERM_BP	GO:0009891	Positive regulation of biosynthetic process	37	9.39	2.02	0.00004
GOTERM_BP	GO:0009893	Positive regulation of metabolic process	46	11.68	1.87	0.00003
GOTERM_BP	GO:0009966	Regulation of signal transduction	46	11.68	1.85	0.00004
GOTERM_BP	GO:0009967	Positive regulation of signal transduction	20	5.08	2.32	0.00044
GOTERM_BP	GO:0010212	Response to ionizing radiation	6	1.52	3.8	0.0048
GOTERM_BP	GO:0010557	Positive regulation of macromolecule biosynthetic process	34	8.63	1.98	0.00011
GOTERM_BP	GO:0010604	Positive regulation of macromolecule metabolic process	44	11.17	1.92	0.00002
GOTERM_BP	GO:0010627	Regulation of protein kinase cascade	15	3.81	2.07	0.00632
GOTERM_BP	GO:0010628	Positive regulation of gene expression	32	8.12	2.11	0.00006
GOTERM_BP	GO:0010646	Regulation of cell communication	50	12.69	1.75	0.00008
GOTERM_BP	GO:0010647	Positive regulation of cell communication	21	5.33	2.25	0.00048
GOTERM_BP	GO:0010740	Positive regulation of protein kinase cascade	13	3.3	2.55	0.00185
GOTERM_BP	GO:0015674	Di-, trivalent inorganic cation transport	13	3.3	2.27	0.00501
GOTERM_BP	GO:0016568	Chromatin modification	18	4.57	2.26	0.00114
GOTERM_BP	GO:0018108	Peptidyl-tyrosine phosphorylation	9	2.28	3.21	0.00197
GOTERM_BP	GO:0018212	Peptidyl-tyrosine modification	9	2.28	3.15	0.00225

Part of GO analysis of biological processes is supplied here; more data can be found in additional files.

**Table 3:** GO analysis of differentially expressed genes associated with the cellular location and molecular function.

Category	GO ID	Term	Count of genes	Percent of count of genes	Fold enrichment	*P* value
GOTERM_CC	GO:0000307	Cyclin-dependent protein kinase Holoenzyme complex	4	0.92	12.35	0.00022
GOTERM_CC	GO:0005576	Extracellular region	82	18.76	1.52	0.00006
GOTERM_CC	GO:0005615	Extracellular space	29	6.64	1.6	0.00908
GOTERM_CC	GO:0005881	Cytoplasmic microtubule	3	0.69	6.95	0.00842
GOTERM_CC	GO:0016324	Apical plasma membrane	10	2.29	2.39	0.00945
GOTERM_CC	GO:0043005	Neuron projection	18	4.12	2	0.00423
GOTERM_CC	GO:0044421	Extracellular region part	41	9.38	1.61	0.00181
GOTERM_CC	GO:0045095	Keratin filament	8	1.83	3.29	0.00297
GOTERM_MF	GO:0000287	Magnesium ion binding	22	5.2	1.73	0.009
GOTERM_MF	GO:0004725	Protein tyrosine phosphatase activity	8	1.89	2.86	0.007
GOTERM_MF	GO:0005057	Receptor signaling protein activity	11	2.6	2.51	0.0047
GOTERM_MF	GO:0005102	Receptor binding	37	8.75	1.55	0.0055
GOTERM_MF	GO:0005104	Fibroblast growth factor receptor binding	4	0.95	6.95	0.0023
GOTERM_MF	GO:0005201	Extracellular matrix structural constituent	7	1.65	3.04	0.0082
GOTERM_MF	GO:0005262	Calcium channel activity	7	1.65	3.32	0.0051
GOTERM_MF	GO:0008083	Growth factor activity	12	2.84	2.72	0.0016
GOTERM_MF	GO:0008138	Protein tyrosine/serine/threonine phosphatase activity	6	1.42	4.86	0.0014
GOTERM_MF	GO:0008168	Methyltransferase activity	11	2.6	2.27	0.0097
GOTERM_MF	GO:0008276	Protein methyltransferase activity	6	1.42	3.77	0.005
GOTERM_MF	GO:0042054	Histone methyltransferase activity	5	1.18	4.05	0.0075

**Table 4:** Ankylosing spondylitis KEGG pathway analysis result.

Group compared	KEGG pathway ID and name	Count of genes	Percent of count of genes	Gene name	*P* value
Normal environment	Hsa04010: MAPK signaling pathway	2	0.30%	CACNA2D3, PPM1B	0.12000
	Hsa04620: toll-like receptor signaling pathway	1	0.15%	TLR6	0.20000

Inflammatory environment	Hsa04010: MAPK signaling pathway	14	0.79%	GNA12, DUSP1, MAPK11, RAC1, MRAS, ELK1, ATF4, MAPK3, CDC25B, STMN1, MAX, TGFBR2, CACNB3, FGF8, and RASA2	0.00000
	Hsa04620: toll-like receptor signaling pathway	8	0.45%	RAC1, TRAF3, MAPK3, IFNAR1, TICAM1, IL6, MAPK11, and IFNA1	0.00002

**Table 5:** Key gene list of signaling pathways related to AS.

Signaling pathway	NCBI gene ID	Gene name	Fold change absolute	*P* value	Regulation	Description
MAPK	1843	DUSP1	3.89	0.02027	Up	Dual specificity phosphatase 1
MAPK	2768	GNA12	3.70	0.02885	Up	Guanine nucleotide binding protein (G protein) alpha 12
MAPK	5879	RAC1	3.15	0.00019	Up	RAS-related C3 botulinum toxin substrate 1 (rho family, small GTP binding protein Rac1)
MAPK	22808	MRAS	3.14	0.00491	Up	Muscle RAS oncogene homolog
MAPK	2002	ELK1	2.80	0.01115	Up	ELK1, member of ETS oncogene family
MAPK	5595	MAPK3	2.73	0.01417	Up	Mitogen-activated protein kinase 3
MAPK	994	CDC25B	2.66	0.03507	Up	Cell division cycle 25B
MAPK	468	ATF4	2.50	0.04995	Up	Activating transcription factor 4 (tax-responsive enhancer element B67)
MAPK	3925	STMN1	2.47	0.01463	Up	Stathmin 1/oncoprotein 18
MAPK	4149	MAX	2.31	0.00410	Up	MYC associated factor X
MAPK	7048	TGFBR2	2.18	0.03642	Up	Transforming growth factor, beta receptor II (70/80 kDa)
MAPK	5600	MAPK11	2.12	0.04901	Up	Mitogen-activated protein kinase 11
MAPK	784	CACNB3	2.04	0.03028	Up	Calcium channel, voltage-dependent, beta 3 subunit
MAPK	2253	FGF8	3.49	0.03611	Down	Fibroblast growth factor 8 (androgen-induced)
MAPK	5922	RASA2	2.83	0.01406	Down	RAS p21 protein activator 2
TLR	7187	TRAF3	2.86	0.03732	Up	TNF receptor-associated factor 3
TLR	3454	IFNAR1	2.37	0.01055	Up	Interferon (alpha, beta, and omega) receptor 1
TLR	148022	TICAM1	2.23	0.02105	Up	Toll-like receptor adaptor molecule 1
TLR	3569	IL6	2.18	0.01526	Up	Interleukin 6 (interferon, beta 2)
TLR	5879	RAC1	3.15	0.00019	Up	RAS-related C3 botulinum toxin substrate 1 (rho family, small GTP binding protein Rac1)
TLR	5595	MAPK3	2.73	0.01417	Up	Mitogen-activated protein kinase 3
TLR	5600	MAPK11	2.12	0.04901	Up	Mitogen-activated protein kinase 11
TLR	3439	IFNA1	2.62	0.03022	Down	Interferon, alpha 1
