# Supplementary material for: Whole Genome Expression Profiling and Signal Pathway Screening of MSCs in Ankylosing Spondylitis
Source: Stem Cells Int. 2014 Dec 3;2014:913050. doi: 10.1155/2014/913050 (PMC4269092; doi:10.1155/2014/913050)
Supplement: Supplementary file 1 — Supplementary Table 1: GO analysis of ankylosing spondylitis with different biological processes related to gene. [file 913050.f1.zip › mat.913050.v1.docx]

**Table 2.** GO analysis of ankylosing spondylitis with different biological processes related to gene.

| Category | GO.ID | Term | Count of genes | Percent of count of genes | Fold.  Enrichment | Pvalue |
| --- | --- | --- | --- | --- | --- | --- |
| GOTERM_BP | GO:0002376 | immune system process | 50 | 12.69 | 1.7 | 0.00016 |
| GOTERM_BP | GO:0002520 | immune system development | 19 | 4.82 | 2.03 | 0.00285 |
| GOTERM_BP | GO:0003006 | reproductive developmental process | 14 | 3.55 | 2.05 | 0.00898 |
| GOTERM_BP | GO:0006325 | chromatin organization | 21 | 5.33 | 1.95 | 0.00276 |
| GOTERM_BP | GO:0006816 | calcium ion transport | 13 | 3.3 | 2.71 | 0.00107 |
| GOTERM_BP | GO:0006950 | response to stress | 64 | 16.24 | 1.35 | 0.00765 |
| GOTERM_BP | GO:0006996 | organelle organization | 54 | 13.71 | 1.4 | 0.00669 |
| GOTERM_BP | GO:0007154 | cell communication | 71 | 18.02 | 1.59 | 0.00005 |
| GOTERM_BP | GO:0007165 | signal transduction | 108 | 27.41 | 1.24 | 0.00756 |
| GOTERM_BP | GO:0007242 | intracellular signaling cascade | 73 | 18.53 | 1.7 | 0 |
| GOTERM_BP | GO:0007243 | protein kinase cascade | 25 | 6.35 | 1.71 | 0.00663 |
| GOTERM_BP | GO:0007275 | multicellular organismal development | 100 | 25.38 | 1.28 | 0.00422 |
| GOTERM_BP | GO:0009891 | positive regulation of biosynthetic process | 37 | 9.39 | 2.02 | 0.00004 |
| GOTERM_BP | GO:0009893 | positive regulation of metabolic process | 46 | 11.68 | 1.87 | 0.00003 |
| GOTERM_BP | GO:0009966 | regulation of signal transduction | 46 | 11.68 | 1.85 | 0.00004 |
| GOTERM_BP | GO:0009967 | positive regulation of signal transduction | 20 | 5.08 | 2.32 | 0.00044 |
| GOTERM_BP | GO:0010212 | response to ionizing radiation | 6 | 1.52 | 3.8 | 0.0048 |
| GOTERM_BP | GO:0010557 | positive regulation of macromolecule biosynthetic process | 34 | 8.63 | 1.98 | 0.00011 |
| GOTERM_BP | GO:0010604 | positive regulation of macromolecule metabolic process | 44 | 11.17 | 1.92 | 0.00002 |
| GOTERM_BP | GO:0010627 | regulation of protein kinase cascade | 15 | 3.81 | 2.07 | 0.00632 |
| GOTERM_BP | GO:0010628 | positive regulation of gene expression | 32 | 8.12 | 2.11 | 0.00006 |
| GOTERM_BP | GO:0010646 | regulation of cell communication | 50 | 12.69 | 1.75 | 0.00008 |
| GOTERM_BP | GO:0010647 | positive regulation of cell communication | 21 | 5.33 | 2.25 | 0.00048 |
| GOTERM_BP | GO:0010740 | positive regulation of protein kinase cascade | 13 | 3.3 | 2.55 | 0.00185 |
| GOTERM_BP | GO:0015674 | di-, tri-valent inorganic cation transport | 13 | 3.3 | 2.27 | 0.00501 |
| GOTERM_BP | GO:0016568 | chromatin modification | 18 | 4.57 | 2.26 | 0.00114 |
| GOTERM_BP | GO:0018108 | peptidyl-tyrosine phosphorylation | 9 | 2.28 | 3.21 | 0.00197 |
| GOTERM_BP | GO:0018212 | peptidyl-tyrosine modification | 9 | 2.28 | 3.15 | 0.00225 |
| GOTERM_BP | GO:0019932 | second-messenger-mediated signaling | 15 | 3.81 | 2.23 | 0.0032 |
| GOTERM_BP | GO:0023046 | signaling process | 119 | 30.2 | 1.21 | 0.00904 |
| GOTERM_BP | GO:0023051 | regulation of signaling process | 46 | 11.68 | 1.85 | 0.00004 |
| GOTERM_BP | GO:0023056 | positive regulation of signaling process | 20 | 5.08 | 2.32 | 0.00044 |
| GOTERM_BP | GO:0023060 | signal transmission | 119 | 30.2 | 1.21 | 0.00896 |
| GOTERM_BP | GO:0030097 | hemopoiesis | 19 | 4.82 | 2.32 | 0.00061 |
| GOTERM_BP | GO:0030154 | cell differentiation | 64 | 16.24 | 1.37 | 0.00517 |
| GOTERM_BP | GO:0030225 | macrophage differentiation | 4 | 1.02 | 8.64 | 0.00097 |
| GOTERM_BP | GO:0031325 | positive regulation of cellular metabolic process | 46 | 11.68 | 1.95 | 0.00001 |
| GOTERM_BP | GO:0031328 | positive regulation of cellular biosynthetic process | 37 | 9.39 | 2.05 | 0.00003 |
| GOTERM_BP | GO:0031401 | positive regulation of protein modification process | 12 | 3.05 | 2.32 | 0.00589 |
| GOTERM_BP | GO:0032268 | regulation of cellular protein metabolic process | 23 | 5.84 | 1.75 | 0.00682 |
| GOTERM_BP | GO:0032270 | positive regulation of cellular protein metabolic process | 14 | 3.55 | 2.15 | 0.00593 |
| GOTERM_BP | GO:0032502 | developmental process | 109 | 27.66 | 1.25 | 0.00538 |
| GOTERM_BP | GO:0035270 | endocrine system development | 7 | 1.78 | 3.96 | 0.00188 |
| GOTERM_BP | GO:0042509 | regulation of tyrosine phosphorylation of STAT protein | 4 | 1.02 | 4.9 | 0.00846 |
| GOTERM_BP | GO:0042517 | positive regulation of tyrosine phosphorylation of Stat3 protein | 3 | 0.76 | 10.02 | 0.00281 |
| GOTERM_BP | GO:0042531 | positive regulation of tyrosine phosphorylation of STAT protein | 4 | 1.02 | 6.39 | 0.00317 |
| GOTERM_BP | GO:0043066 | negative regulation of apoptosis | 19 | 4.82 | 1.89 | 0.00622 |
| GOTERM_BP | GO:0043069 | negative regulation of programmed cell death | 19 | 4.82 | 1.86 | 0.00714 |
| GOTERM_BP | GO:0043086 | negative regulation of catalytic activity | 16 | 4.06 | 2.15 | 0.00337 |
| GOTERM_BP | GO:0044092 | negative regulation of molecular function | 18 | 4.57 | 1.99 | 0.00455 |
| GOTERM_BP | GO:0044093 | positive regulation of molecular function | 26 | 6.6 | 1.65 | 0.00889 |
| GOTERM_BP | GO:0045649 | regulation of macrophage differentiation | 3 | 0.76 | 10.02 | 0.00281 |
| GOTERM_BP | GO:0045893 | positive regulation of transcription, DNA-dependent | 24 | 6.09 | 1.94 | 0.00158 |
| GOTERM_BP | GO:0045935 | positive regulation of nucleobase, nucleoside, nucleotide and nucleic acid metabolic process | 32 | 8.12 | 1.96 | 0.00023 |
| GOTERM_BP | GO:0045941 | positive regulation of transcription | 30 | 7.61 | 2.05 | 0.00016 |
| GOTERM_BP | GO:0046427 | positive regulation of JAK-STAT cascade | 4 | 1.02 | 5.65 | 0.00503 |
| GOTERM_BP | GO:0046578 | regulation of Ras protein signal transduction | 13 | 3.3 | 2.15 | 0.00789 |
| GOTERM_BP | GO:0048513 | organ development | 68 | 17.26 | 1.42 | 0.00181 |
| GOTERM_BP | GO:0048518 | positive regulation of biological process | 78 | 19.8 | 1.44 | 0.00047 |
| GOTERM_BP | GO:0048522 | positive regulation of cellular process | 72 | 18.27 | 1.46 | 0.00059 |
| GOTERM_BP | GO:0048523 | negative regulation of cellular process | 62 | 15.74 | 1.37 | 0.00605 |
| GOTERM_BP | GO:0048534 | hemopoietic or lymphoid organ development | 19 | 4.82 | 2.16 | 0.0014 |
| GOTERM_BP | GO:0048731 | system development | 84 | 21.32 | 1.3 | 0.00514 |
| GOTERM_BP | GO:0048856 | anatomical structure development | 89 | 22.59 | 1.27 | 0.00883 |
| GOTERM_BP | GO:0048869 | cellular developmental process | 66 | 16.75 | 1.34 | 0.00825 |
| GOTERM_BP | GO:0050731 | positive regulation of peptidyl-tyrosine phosphorylation | 5 | 1.27 | 3.83 | 0.00954 |
| GOTERM_BP | GO:0050790 | regulation of catalytic activity | 42 | 10.66 | 1.82 | 0.00012 |
| GOTERM_BP | GO:0051056 | regulation of small GTPase mediated signal transduction | 15 | 3.81 | 2.12 | 0.00515 |
| GOTERM_BP | GO:0051173 | positive regulation of nitrogen compound metabolic process | 34 | 8.63 | 2.01 | 0.00009 |
| GOTERM_BP | GO:0051246 | regulation of protein metabolic process | 25 | 6.35 | 1.66 | 0.00903 |
| GOTERM_BP | GO:0051247 | positive regulation of protein metabolic process | 14 | 3.55 | 2.07 | 0.00812 |
| GOTERM_BP | GO:0051254 | positive regulation of RNA metabolic process | 24 | 6.09 | 1.92 | 0.00177 |
| GOTERM_BP | GO:0051276 | chromosome organization | 24 | 6.09 | 1.74 | 0.00635 |
| GOTERM_BP | GO:0051302 | regulation of cell division | 5 | 1.27 | 3.99 | 0.00798 |
| GOTERM_BP | GO:0051646 | mitochondrion localization | 3 | 0.76 | 9.19 | 0.00367 |
| GOTERM_BP | GO:0051781 | positive regulation of cell division | 5 | 1.27 | 4.83 | 0.00349 |
| GOTERM_BP | GO:0060548 | negative regulation of cell death | 19 | 4.82 | 1.84 | 0.00796 |
| GOTERM_BP | GO:0065009 | regulation of molecular function | 49 | 12.44 | 1.84 | 0.00002 |
| GOTERM_BP | GO:0070838 | divalent metal ion transport | 13 | 3.3 | 2.67 | 0.00125 |
