# Supplementary material for: Whole Genome Expression Profiling and Signal Pathway Screening of MSCs in Ankylosing Spondylitis
Source: Stem Cells Int. 2014 Dec 3;2014:913050. doi: 10.1155/2014/913050 (PMC4269092; doi:10.1155/2014/913050)
Supplement: Supplementary file 1 — Supplementary Table 1: GO analysis of ankylosing spondylitis with different biological processes related to gene. [file 913050.f1.zip › suppl. description.docx]

**Additional File 1.** GO analysis of ankylosing spondylitis with different biological processes related to gene.

Bone marrow MSCs were isolated from the bone marrow aspirates by density gradient centrifugation. Then, MSCs were cultured in two different conditions: in normal media (Norm) and MSCs cultured in an inflammatory environment (Infla) which was created by adding TNF-α (10ng/ml) and IFN-γ (10ng/ml) into normal culture media for four hours. Total RNA of cultured MSCs were extracted and then sent to execute whole genome expression analysis. Scanned images were then imported into NimbleScan software (version 2.5) for grid alignment and expression data analysis. Probe level files and gene level files were generated after normalization. Differentially expressed genes were identified using Volcano Plot filtering. KEGG Pathway Analysis and GO analysis were applied to determine the roles of these differentially expressed genes in biological pathways or GO terms. Finally, the post hoc Bonferroni Test was used to correct for multiplicity since a large number of false positive p-values were observed. P values *< 0.01* were considered significant. Here provides the total results of GO analysis of ankylosing spondylitis with different biological processes related to gene.
